# Diversity Patterns and Drivers of Soil Bacterial and Fungal Communities in a Muddy Coastal Wetland of China

**DOI:** 10.3390/jof10110770

**Published:** 2024-11-06

**Authors:** Baohan Song, Tianyi Wang, Cheng Wan, Yuan Cai, Lingfeng Mao, Zhiwei Ge, Nan Yang

**Affiliations:** Co-Innovation Center for Sustainable Forestry in Southern China, Laboratory of Biodiversity and Conservation, College of Ecology and Environment, Nanjing Forestry University, 159 Longpan Road, Nanjing 210037, China; songbh@njfu.edu.cn (B.S.); 8221711750@njfu.edu.cn (T.W.); wancheng@njfu.edu.cn (C.W.); caiyuan2023@njfu.edu.cn (Y.C.); maolingfeng2008@163.com (L.M.)

**Keywords:** coastal wetland, soil microbial community, vegetation types, soil properties

## Abstract

Elucidating the dynamics of soil microbial diversity in coastal wetlands is essential for understanding the changes in ecological functions within these ecosystems, particularly in the context of climate change and improper management practices. In this study, the diversity patterns and influencing factors of soil bacterial and fungal communities in a muddy coastal wetland in China were investigated using Illumina sequencing of 16S rRNA and ITS1, across wetlands dominated by different vegetations and varying proximity to the coastline. The wetlands include four plots dominated by *Spartina alterniflora* (SA1), four plots dominated by *Suaeda glauca* (SG2), additional four plots of *Suaeda glauca* (SG3), and four plots dominated by *Phragmites australis* (PA4), ranging from the nearest to the coast to those farther away. The results revealed significant differences in bacterial richness (Observed_species index) and fungal diversity (Shannon index) across different wetlands, with SG3 demonstrating the lowest bacterial Observed_species value (1430.05), while SA1 exhibited the highest fungal Shannon value (5.55) and PA4 showing the lowest fungal Shannon value (3.10). Soil bacterial and fungal community structures differed significantly across different wetlands. The contents of soil available phosphorus and total phosphorus were the main drivers for fungal Observed_species and Shannon index, respectively. Soil organic carbon, pH, and salinity were indicated as the best predictors of bacterial community structure, accounting for 28.1% of the total variation. The total nitrogen content and soil salinity contributed mostly to regulating fungal community structure across different wetlands, accounting for 19.4% of the total variation. The results of this study offer a thorough understanding of the response and variability in soil microbial diversity across the muddy coastal wetlands in China.

## 1. Introduction

As the intermediary region connecting terrestrial and marine ecosystems, coastal wetlands effectively perform essential and distinctive ecological functions in nutrients cycling, erosion control, pollutants purification, and biodiversity maintenance [1]. However, the rising sea level and shifting vegetation caused by climate change and improper management practices, have the potential to influence the biodiversity of coastal wetlands, thus posing a threat to the integrity of soil-vegetation ecosystems. The dynamics of soil microbial diversity can offer valuable insights into the changes in the functionality, health, and stability of coastal wetland ecosystems [2,3]. Therefore, it is essential to investigate the diversity patterns of soil microbial diversity in coastal wetlands, to better preserve the wetland ecosystems and predict the influence of the changing environments.

Soil microorganisms demonstrate significant sensitivity to fluctuations in both abiotic and biotic conditions [4,5]. From one perspective, soil microbial diversity in coastal wetland ecosystems may fluctuate in response to variations in soil physicochemical properties, that are influenced by the proximity to the coastline [6,7]. Corresponding to the proximity of different wetlands to the coastline, previous studies have shown that soil salinity is a critical determinant in shaping the microbial diversity in coastal wetland environments [2,8]. Previous studies indicated soil microbial diversity declined significantly with increasing levels of soil salinity [9,10], the elevation in salinity levels may diminish the accessibility of soil organic matter to the soil microbial community [11]. While other studies suggested soil pH as an ideal indicator for monitoring soil bacterial diversity in coastal wetlands [12,13], soil pH decreased with the distance to the coastline and drove the shifts in bacterial community structure [14]. Additionally, soil water content was considered as another important factor influencing microbial diversity. Variations in wetland hydrology facilitate a dynamic transition between aerobic and anaerobic conditions, thereby influencing the composition and functionality of microbial communities in wetland soils [15].

From another perspective, soil microbial diversity in coastal wetlands was reported to be predominantly shaped by vegetation type [1]. The soil carbon and nutrients input are dependent on the specific composition of the litter or root exudates produced by various plant communities [16,17], soil microbes may exhibit different responses to diverse inputs and reside in the rhizosphere under specific soil conditions [18]. Consequently, the evolving distribution of natural plant communities in wetland ecosystems may modify soil properties, thereby impacting the diversity and composition of soil microbial communities [18]. However, the extent and mechanisms of alterations in plant communities resulting from biological invasion may influence soil microbial diversity in wetlands remain inclusive and need further investigation.

Furthermore, soil bacteria and fungi may exhibit contrasting diversity patterns in response to environmental variables in coastal wetlands, due to their differentiations [19]. A recent study indicated bacterial community exhibited a higher degree of sensitivity to variations in soil salinity and soil pH, as opposed to the fungal community [20,21]. While fungi have a more intimate relationship with plants in general and exhibit a greater degree of reliance on the resources or exudates supplied by plants (e.g., mycorrhizal fungi) [22]. Therefore, illustrating the different diversity patterns of bacterial and fungal communities in different coastal wetlands may help to further clarify the underlying determinants of microbial diversity in coastal wetlands.

However, most current research was focused on exploring the microbial diversity in estuarine wetland ecosystems (such as the Yellow River Delta wetland) [9,23], there remains a notable deficiency in the studies pertaining to muddy coastal wetland ecosystems. Muddy wetlands typically possess broad tidal flats and the soil consists primarily of silt, rendering it exceptionally fertile [24]. The distribution of the vegetation community is primarily influenced by the frequency of tidal incursion and the soil salt content, with halophytes being dominant [25]. This study was conducted in the largest muddy coastal wetland in China, the Tiaozini coastal wetland, which harbors abundant biodiversity and provides important ecological functions [26]. The Tiaozini coastal wetland historically features an abundance of *Phragmites australis* and *Suaeda glauca*. Following the introduction of *Spartina alterniflora* for the purpose of beach fortification, there has been a noticeable transition towards *Spartina alterniflora* as the predominant plant species within the coastal wetlands in recent years. However, whether the alterations in plant communities in the Tiaozini coastal wetland will affect soil microbial diversity remains unclear. Therefore, this study undertook an investigation into the diversity patterns and influencing factors of soil bacterial and fungal communities within the wetlands, characterized by varying dominant vegetation and differing distances from the coastline in the Tiaozini coastal wetland. This investigation will contribute to our understanding of the effects of rising sea levels and changing vegetation on wetland ecosystems. The following was hypothesized: (1) the shifting vegetations had a notable impact on the variety of soil microbial diversities across different wetlands; (2) soil salinity was the primary determinant to regulate the variations in soil microbial diversities in coastal wetlands; (3) after soil salinity, soil pH was the primary regulatory factor of bacterial diversity, and nutrients input under different vegetations predominantly determining fungal diversity.

## 2. Materials and Methods

### 2.1. Site Description and Sample Collection

**Figure 1:**
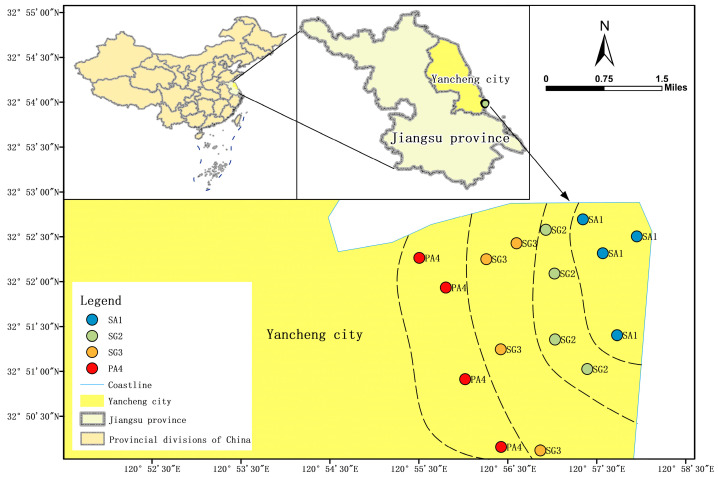
Overview of the sampling points and overall sampling location. SA1, plots of *Spartina alterniflora*; SG2, plots of *Suaeda glauca*; SG3, additional plots of *Suaeda glauca*; PA4, plots of *Phragmites australis*.

The study was conducted in the Tiaozini coastal wetland (32°43′53″–32°52′57″ N, 120°53′58″–121°3′13″ E) (Figure 1). This area is recognized as a representative muddy wetland situated along the eastern coast of China. The mean annual temperature and mean annual precipitation range from 13.7 to 14.8 °C and 800 to 1100 mm, respectively. The soil is classified as silt soil, according to the World Reference Base [27]. The vegetation composition in the coastal wetland exhibits significant variability based on the proximity to the coastline, *Spartina alterniflora* predominates in close proximity to the coastline, followed by *Suaeda glauca* which occupies the intermediate zone between *Spartina alterniflora* and *Phragmites australis*.

In August 2023, sampling plots dominated by different vegetations were randomly selected. The plots near and far from the coastline were selected: four plots of *Spartina alterniflora* (SA1), four plots of *Suaeda glauca* (SG2), an additional four plots of *Suaeda glauca* (SG3), and four plots of *Phragmites australis* (PA4) (Figure 1). Each plot (20 m × 20 m) was spaced approximately 300 m apart from the neighboring plots. Five sampling points in each plot were randomly chosen, to collect soil samples (0–10 cm after litter removal) by soil cores, and merged into one single sample. In total, 16 soil samples were collected (4 vegetation types × 4 plots = 16). Subsequently, the fresh soil samples were sifted (2 mm sieve after sterilization) and divided into three aliquots. One aliquot was used for molecular microbial analysis and stored in a 10 mL sterile centrifuge tube at −80 °C. The second aliquot was used to analyze the contents of soil ammonium (NH_4_^+^), nitrate (NO_3_^−^), and available phosphorus (AP), and stored at −20 °C. The remaining aliquot was air-dried at room temperature and was stored at 4 °C to determine the soil pH, conductivity, total nitrogen (TN), total phosphorus (TP), and soil organic carbon (SOC).

### 2.2. Soil Physiochemical Properties

After drying the fresh soil at 105 °C for 24 h, the soil water content (MC) was determined using the gravimetric method. Using 2 mol/L potassium chloride (KCL) as the extract, UV spectrophotometry was used to determine the soil NO_3_^−^ and NO_4_^+^ content [28]. AP was analyzed colorimetrically after extraction and fully reacted with molybdenum and antimony resistance. TP was analyzed colorimetrically after sulfuric acid-perchloric acid (H_2_SO_4_-HClO_4_) digestion (Agilent Cary 100, Santa Clara, CA, USA) [29]. Soil pH and soil conductivity were measured using a pH meter (LA-pH10, Hach, Loveland, CO, USA) and a conductivity meter (DDS-307A, Shanghai, China) [30,31]. Soil TN was quantified using a CN-element analyzer (PE2400II, Perkinelmer, Waltham, MA, USA) [32]. After oxidizing the organic matter in the soil with a potassium dichromate solution, SOC was determined by using a spectrophotometer [33].

### 2.3. Extraction and Amplification of Soil Microbial DNA

DNA was extracted from the 16 soil samples using the MagBeads FastDNA kit for Soil (MP Biomedicals, Santa Ana, CA, USA), according to the manufacturer’s instructions, and kept at a temperature of −20 °C for PCR amplifications. The bacterial V4–V5 region was amplified by employing the primers 515F (5′-GTGCCAGCMGCCGCGGTAA-3′) and 907R (5′-CCGTCAATTCMTTTRAGTTT-3′) primer pair [34]. The fungal ITS1 region was amplified using ITS3 (5′-GCATCGATGAAGAACGCAGC-3′) and ITS4 (5′-TCCTCCGCTTATTGATATGC-3′) [35]. The specific methods of PCR thermal cycling followed that described by Yang et al. [36]. Then, the amplicons underwent the processes of extraction, purification, and quantification, and were subjected to paired-end sequencing on an Illumina MiSeq platform (MiSeq-PE250, Personalbio, Shanghai, China).

### 2.4. Illumina Miseq Sequencing

The raw sequence data were processed with QIIME2 2022.11 platform [37], after trimming the primer sequences with the cutadapt plugin (v2.3). This was followed by the quality filtering, denoising, merging, and removal of chimeras from the remaining sequences with the DADA 2 plugin [38], and non-singleton amplicon sequence variants (ASVs) clustering were aligned using mafft [39]. A total of 32,233-16S RNA ASVs and 1856-ITS ASVs were used for further analysis. Rarefaction and alpha diversity (Observed_species richness and Shannon diversity index) of ASVs were performed on resampled datasets of 57,925 16S RNA sequences and 64,800 ITS sequences (minimum sample sequence) per soil sample, to standardize the samples. UCLUST was used to assign taxonomy with the SILVA database (Release132, http://www.arb-silva.de, accessed on 1 September 2024) for 16S RNA ASVs [40] and BLAST was utilized to classify taxonomy using the UNITE database (Release 8.0, https://unite.ut.ee/, accessed on 1 September 2024) for ITS ASVs [41]. The Naive Bayes scikit-learn classifier was employed to perform multi-class taxonomy classification of ITS sequences and 16S rRNA in QIIME 2, and the Naive Bayes parameters were set as follows: k-mer lengths of 7 and confidence = 0.94 (bacteria); k-mer lengths of 7 and confidence = 0.98 (fungi) [42]. Initial sequences are available in the NCBI Sequence Read Archive under Bioproject PRJNA1072589, under accession numbers SRR27844171-SRR27844186 (bacteria) and SRR27883384-SRR27883399 (fungi).

### 2.5. Statistical Analysis

Shapiro–Wilk [43] and Levene [44] tests were executed to assess the normality and homogeneity of the data prior to conducting any statistical analysis. Analysis of variance (ANOVA) and Tukey’s post hoc tests were conducted to test for variations in soil properties and soil microbial alpha-diversity indices across different wetlands [45].The Pearson’s correlation analysis was utilized to examine the relationships between soil properties and microbial alpha-diversity [46]. The nonparametric statistical test, the Kruskal–Wallis test was used to compare the relative abundance of dominant bacteria and fungi at phylum and genus levels across different wetlands [47], while Spearman’s rank correlation analysis was used to analyze the relationship between soil properties and the relative abundance of dominant bacterial and fungal phyla and genera [48].

The optimal indicators of soil microbial alpha-diversity indices were elucidated through a multivariate linear regression model utilizing Akaike’s information criteria (AIC), employing the “mass” package in R [49,50]. Analysis of similarities (ANOSIM) was used to analyze the distance between the microbial communities (based on Bray–Curtis distance matrix method), and canonical correlation analysis (CCA) was conducted to test the influences of soil properties on the community compositions of bacteria and fungi (ASV level) [51]. All mathematical statistical analysis was performed by R 4.2.0.

## 3. Results

### 3.1. Soil Properties

The soil properties exhibited notable variations across different wetlands in relation to the proximity to the coastline (Table 1). The contents of soil moisture (MC), ammonia (NH_4_^+^), nitrate (NO_3_^−^), soil organic carbon (SOC), and total nitrogen (TN) were significantly higher in the *Spartina alterniflora* (SA1) plot, which was closest to the coastline, compared to the other plots (*p* < 0.001). The contents of total phosphorus (TP) notably decreased with increasing distance from the coastline, which was significantly higher in the SA1 and *Suaeda glauca* (SG2) plots compared to the SG3 and *Phragmites australis* (PA4) plots (*p* < 0.001). In contrast, soil conductivity exhibited the highest value in the SG2 plots, located in the second closest distance to the coastline after SA1, then decreased with increasing distance from the coastline, with the lowest value in the PA4 plots locating the farthest distance from the coastline. Furthermore, soil pH did not follow a pattern correlating with the proximity to the coastline, with the lowest soil pH observed at the SG3 plots, and the highest soil pH at SG2 plots (*p* < 0.001).

### 3.2. Soil Microbial Alpha-Diversity

Among the 16 soil samples, a total of 1,346,793 high-quality sequences of bacterial communities and 1,462,340 fungal communities were obtained. The number of refined sequence readings per sample ranged from 57,925 to 121,944 for bacteria (with a mean of 84,175) and 64,800 to 140,694 for fungi (with a mean of 91,396). In terms of the read lengths attributed to the 16S rRNA and ITS gene, which varied from 237 to 442 base pairs of bacteria (with a mean of 387 bp) and 123 to 439 base pairs of fungi (with a mean of 296 bp). Moreover, the Goods_coverage values for each soil sample demonstrated consistently high levels exceeding 99% for both bacteria and fungi, elucidating the high reliability of the sequencing accuracy.

**Figure 2:**
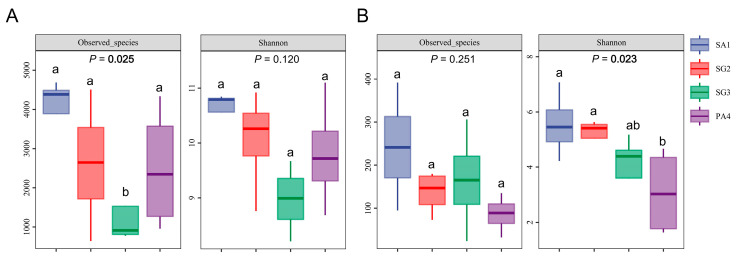
Alpha-diversity indices of bacteria (**A**) and fungi (**B**) across different wetlands in the Tiaozini coastal wetland. Alpha diversity indices were based on ASVs richness (Observed_species index) and diversity (Shannon index). SA1, plots of *Spartina alterniflora*; SG2, plots of *Suaeda glauca*; SG3, additional plots of *Suaeda glauca*; PA4, plots of *Phragmites australis*. Different letters indicate significant differences at *p* < 0.05 (ANOVA) between means (*n* = 4).

The soil bacterial Observed_species index exhibited variations ranging from 1430.05 to 3994.15, and the Shannon index demonstrated a range of variability between 8.97 and 10.59 across different wetlands (Figure 2A, Table S1). The lowest Observed_species index was detected at the SG3 plots (*P*_Observed_species_ = 0.025), while no significant variation in the bacterial Shannon indices was observed across the wetlands dominated by different vegetations (*P*_Shannon_ = 0.120, Figure 2A, Table S1). Pearson test indicated that soil MC, TC, and TN were positively correlated with the bacterial Observed_species index (*P*_MC_ = 0.049, *P*_TC_ = 0.044, *P*_TN_ = 0.036, Table 2).

Soil fungal Observed_species index varied from 86.58 to 242.38, with no significant variations in the index being observed across the different wetlands (*P*_Observed_species_ = 0.251, Figure 2B, Table S1). And soil fungal Shannon index exhibited a significant decrease in correlation to the distance from the coastline, with the highest value observed in SA1 (5.55), and the lowest value in PA4 (3.10) (*P*_Shannon_ = 0.023, Figure 2B, Table S1). Soil fungal Observed_species index demonstrated a positive correlation with AP (*P*_AP_ = 0.035, Table 2), and soil fungal Shannon index exhibited a positive correlation with TP and conductivity (*P*_TP_ = 0.011, *P*_conductivity_ = 0.022, Table 2).

Soil MC was identified as the most effective indicator in predicting bacterial Observed_species index (Table 3). Soil phosphorus was important in regulating fungal alpha-diversity, with soil AP and TP (RI = 0.336) as the best predictors for the variations in fungal Observed_species index and fungal Shannon index, respectively. This was followed by the conductivity (RI = 0.269), which was also indicated as a good indicator of soil fungal Shannon index in the studied coastal wetland (Table 3).

### 3.3. Soil Microbial Community Structure

**Figure 3:**
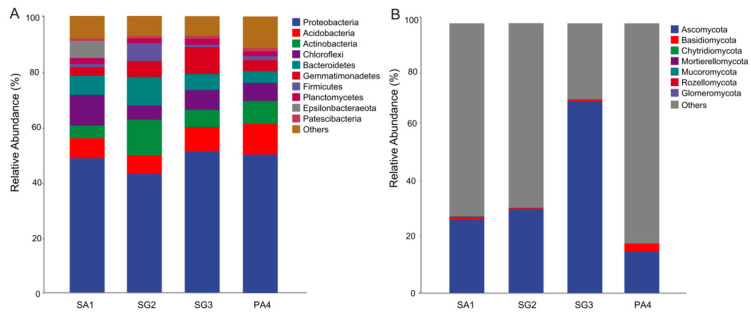
Soil bacterial (**A**) and fungal (**B**) community compositions (at phylum level) across different wetlands in the Tiaozini coastal wetland. SA1, plots of *Spartina alterniflora*; SG2, plots of *Suaeda glauca*; SG3, additional plots of *Suaeda glauca*; PA4, plots of *Phragmites australis*.

For bacteria, Proteobacteria, Acidobacteria, Actinobacteria, Chloroflexi, Bacteroidetes, and Gemmatimonadetes together accounted for 81.5–88.8% of the total sequences across the wetlands dominated by vegetation (with a relative abundance exceeding 5% on average) (Figure 3A, Table S2). Among these, bacterial phylum Proteobacteria demonstrated high prevalence, with a relative abundance exceeding 40%, which exhibited the highest relative abundance (51.3%) in SG3, and the lowest abundance (42.9%) in SG2. The relative abundance of Proteobacteria in SG2 had a statistical difference compared with PA4 (*p* = 0.031), while Firmicutes in SG2 had a statistical difference compared with SG3 (*p* = 0.024, Table S2). The relative abundance of Chloroflexi (*p* = 0.010) and Epsilonbacteraeota (*p* = 0.022) decreased significantly with increasing distance from the coastline. At the genus level, several bacterial genera (*NB1-j*, *Subgroup_10*, *BD2-11_terrestrial_group*, *Woeseia*) were abundant across wetlands (with the mean relative abundance > 2%) (Table S3). Among these, the relative abundance of *NB1-j* increased significantly with the increasing distance from the coastline (PA4 had statistical difference compared with SA1, *p* = 0.014). But the relative abundance of *Woeseia* was significantly higher in SA1, which was closest to the coastline, compared to SG2 (*p* = 0.020).

For fungi, Ascomycota accounted for 15.5–71.2% of the total sequences across the wetlands dominated by different vegetation (with a relative abundance exceeding 5% on average), followed by the Basidiomycota, Chytridiomycota, and Mortierellomycota (with a lower relative abundance < 5%) (Figure 3B, Table S2). The relative abundance of Ascomycota in SG3 had a statistical difference compared with PA4 (*p* = 0.016, Table S2). At the genus level, three genera with the highest relative abundance were *Alternaria*, and *Lignincola* (with the mean relative abundance > 2%) (Table S3). Among these, the relative abundance of *Lignincola* in SG3 had a statistical difference compared with SA1 (*p* = 0.013), while *Phaeosphaeria* in PA4 had a statistical difference compared with SA1 (*p* = 0.014).

### 3.4. Environmental Determinants of Soil Microbial Community Structures

**Figure 4:**
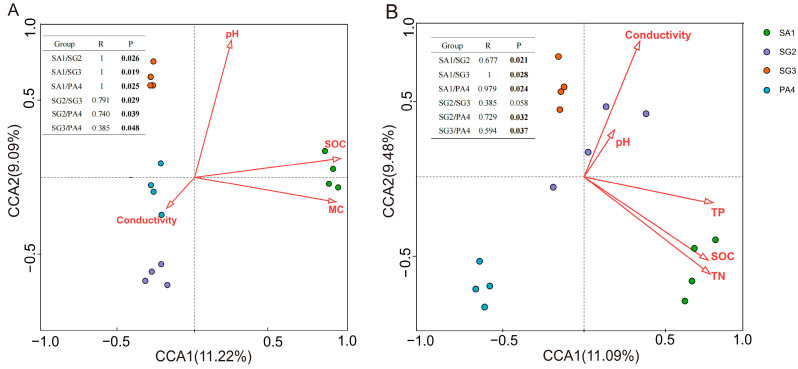
Canonical correlation analysis (CCA) of soil bacterial (**A**) and fungal (**B**) communities across different wetlands in the Tiaozini coastal wetland (*n* = 24). Soil bacterial and fungal community composition was analyzed by ANOSIM using Bray–Curtis as the similarity measure. *p*-Values and R-values after sequential Bonferroni correction are indicated. Significant *p*-values (<0.05) are indicated in bold letters.

Canonical correlation analysis (CCA) and analysis of similarities (ANOSIM) showed that the soil bacterial and fungal community structures were both clearly separated by the plots of different vegetation, respectively (*P_bacteria_* < 0.001, *P_fungi_* < 0.001, Figure 4). CCA indicated soil SOC, pH, and conductivity significantly affected the soil bacterial community structures (*P*_SOC_ = 0.002, *P*_pH_ = 0.008, *P*_conductivity_ = 0.024, Figure 4A, Table S4), which explained 11.1%, 8.8%, and 8.2% of the total variations in bacterial community structures, respectively (Figure 4A, Table S4). For fungi, soil TN and conductivity significantly correlated with soil fungal community structures across the wetlands of different vegetation (*P*_TN_ = 0.002, *P*_conductivity_ = 0.002, Figure 4B, Table S5), which explained 10.3% and 9.1% of the total variations in fungal community structures, respectively (Figure 4B, Table S5).

**Figure 5:**
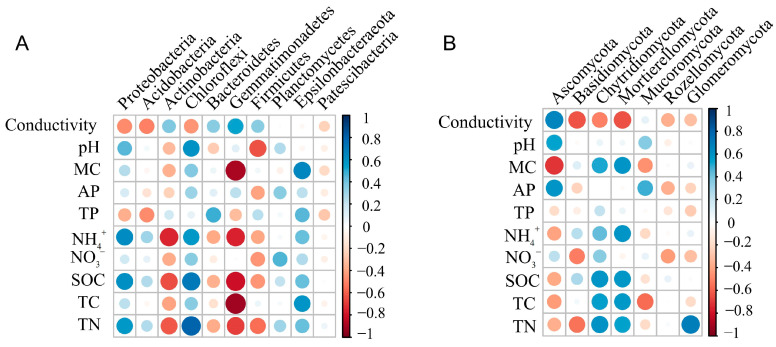
Spearman correlations between the main bacterial (**A**) and fungal (**B**) phyla and soil properties across different wetlands in the Tiaozini coastal wetland.

For bacterial communities, soil MC was positively correlated with the relative abundance of Epsilonbacteraeota (*p* = 0.006), while negatively correlated with the relative abundance of Gemmatimonadetes (*p* < 0.001). The relative abundance of Acidobacteria showed negative relationships with conductivity (*p* = 0.048). The relative abundance of Actinobacteria showed negative relationships with NH_4_^+^ and SOC (*P*_NH4_^+^ < 0.001, *P*_SOC_ = 0.007). Congruently, soil NH_4_^+^, SOC, and TN positively affected the relative abundance of Proteobacteria and Chloroflexi (*p* < 0.05). Conversely, soil NH_4_^+^ was negatively correlated with Gemmatimonadetes (*p* < 0.001) (Figure 5A, Table S6). At the genus level, soil MC was positively correlated with the relative abundance of *Sulfurovum* (*p* = 0.004), while negatively correlated with the relative abundance of *Subgroup_10* (*p* = 0.007) and *BD2-11_terrestrial_group* (*p* < 0.001). The relative abundance of *NB1-j* showed negative relationships with TP (*p* = 0.006) and TC (*p* = 0.046). In addition, the relative abundance of *Woeseia* was positively correlated with AP, SOC and TN (*P*_AP_ = 0.027, *P*_SOC_ = 0.034, *P*_TN_ = 0.004, Table S7).

For fungi, the conductivity, pH, and AP were positively correlated with Ascomycota (*P*_Conductivity_ = 0.005, *P*_pH_ = 0.032, *P*_AP_ = 0.015). Soil MC, SOC, TC, and TN were positively correlated with the relative abundance of Chytridiomycota (*P*_MC_ = 0.036, *P*_SOC_ = 0.015, *P*_TC_ = 0.026, *P*_TN_ = 0.013). Furthermore, the relative abundance of Mucoromycota showed positive relationships with the AP (*p* = 0.049) (Figure 5B, Table S6). At the genus level, the conductivity was positively correlated with the relative abundance of *Hortaea* (*p* = 0.029) and *Macrophoma* (*p* = 0.030). Soil MC, AP, SOC, and TC were positively correlated with the relative abundance of *Lignincola* (*P*_MC_ = 0.007, *P*_AP_ < 0.001, *P*_SOC_ = 0.037, *P*_TC_ < 0.001). TP and TC were positively correlated with the relative abundance of *Phaeosphaeria* (*P*_TP_ < 0.001, *P*_TN_ = 0.028, Table S7).

## 4. Discussion

### 4.1. Variations in Soil Properties Across Different Wetlands

Geographical location and plant characteristics determine the heterogeneity of soil properties within a particular area [17]. Our results indicated significant variations in soil properties across the wetlands (Table 1). Generally, soil in close proximity to the coastline tends to have higher moisture levels due to the influence of subterranean water levels [15], which is the case in our study that the highest soil moisture content (MC) was found in *Spartina alterniflora* community (SA1) (Table 1). Meanwhile, the higher leaf area coefficient and cover rate of the *Spartina alterniflora* community could lead to a reduction in soil water evaporation [52]. In contrast, the soil MC of the *Phragmites australis* community (PA4) located at a greater distance from the coastline exceeded that of the *Suaeda glauca* community (SG2 and SG3). This could be attributed to the root system of *Phragmites australis*, which possessed the capacity for hydraulic lifting. This process facilitated the elevation of groundwater to the surface, allowing for enhanced absorption and utilization by roots [53].

Soil salinity is influenced by various factors such as a capillary rise in saline groundwater and intrusion of saline water from the sea [54], which normally positively correlates with the proximity to the coastline [55]. This is consistent with our results that higher salinity was observed closer to the coastline, except for SA1, the soil conductivity of SA1 was lower than that of SG2 (Table 1). One possible explanation for this disparity is the halophytic nature of *Spartina alterniflora*, which enabled its roots to effectively uptake excessive salt levels in the soils [56]. Additionally, the higher soil MC in *Spartina alterniflora* community acted to dilute the soil salinity, which may also be a contributing factor.

The relationship between soil pH and the proximity to the coastline did not display a discernible pattern. Zhao et al. [57] found that soil salinity and soil pH generally exhibit collinearity in alkaline soils. In this study, there was no significant correlation between soil salinity levels and soil pH levels. It is speculated that the correlation between soil pH and salt levels may be constrained by the range of salinity present. Furthermore, the elevated salinity levels found in the Tiaozini wetland are believed to disrupt this linear relationship [58]. The pH range of the soil was 7.90–8.69, indicating a slightly alkaline nature of the wetland soil, aligning with the typical attributes observed in coastal wetlands [59].

Our results showed that SA1 plots exhibited the highest contents of soil nutrients, which was dominated by *Spartina alterniflora*. Specifically, the levels of carbon, nitrogen, and phosphorus in SA1 plots surpassed those of the other plots (Table 1). The reason could be attributed to the influence of plant litter and root activity on nutrient bio-geochemical cycling [60]. A variety of compounds are released from root exudate and litter decomposition, resulting in an enhancement of soil nutrient levels [61]. In addition, the exudates from halophytes have been found to promote soil microbial growth and enzyme activity, thereby accelerating the decomposition of detritus [62]. In the studied wetlands, the strong stress tolerance of *Spartina alterniflora* resulted in a higher level of litter and root biomass, compared with *Suaeda glauca* and *Phragmites australis* [63]. As a result of this increased input of resources into the soil, there was a noticeable enhancement in the availability of soil nutrients.

### 4.2. Drivers of Soil Bacterial Diversity Across Different Wetlands

Bacteria overwhelmingly dominate the soil microorganism community and play a crucial role in nutrient cycling within wetland ecosystems [64]. In this study, soil MC was suggested as a good indicator to predict bacterial diversity (Table 3). The reason is that the movement of bacteria within the soil environment and the process of nutrient uptake are dependent on the dynamics of the water film in the soil [65]. Therefore, variations in water conditions exerted a more substantial influence on bacteria as opposed to fungi. In addition, water availability is related to the extracellular enzyme activity of soil microorganisms, and water affects microbial metabolic activity by adjusting carbon and other nutrient distribution [66].

The dominant bacterial phylum across the studied wetlands was Proteobacteria (Figure 3A, Table S2), followed by Acidobacteria and Actinobacteria. This was consistent with previous studies, suggesting that Proteobacteria demonstrate strong adaptabilities to various environments, often establishing dominance within bacterial communities [67,68]. Zeng et al. [69] proposed that soil bacterial communities exhibited a consistent composition at the phylum level, irrespective of variations in land use or ecosystem type. The predominant bacterial taxa identified in a vast majority of soil samples included Proteobacteria, Actinobacteriota, Acidobacteriota, and Chloroflexi. Their findings indicate that although soil bacterial community structure varied at a regional scale, several dominant taxa remained consistent across different locations.

In this study, the content of soil organic carbon (SOC) was suggested as the most important determinant of soil bacterial community structure across the wetlands (Figure 4A, Table S4), which was contrary to our second hypothesis. It is in accordance with Delgado et al. [70], who identified that the SOC gradient played a crucial role in determining bacterial distribution patterns on a global scale through meta-analyses. The predominant proportion of soil bacteria rely on the breakdown of organic material for the maintenance of their energy reserves, which are highly responsive to changes in SOC [71,72]. Specifically, the relative abundances of Proteobacteria (*Woeseia*, *Sva1033*, *Subgroup_21*, *SEEP-SRB1*), Chloroflexi (*SBR1031*), and Epsilonbacteraeota (*Sulfurovum*) were positively correlated with SOC (Figure 5A, Tables S6 and S7). Therefore, their relative abundances were the highest in *Spartina alterniflora* communities with higher SOC content.

Aside from SOC, pH emerged as another important factor influencing the structure of bacterial communities (Figure 4A, Table S4). This was in alignment with previous studies, which have identified pH as the primary predictor for soil bacterial community composition [22,73]. The variation range of soil pH in this study was between 7.90 and 8.69 (Table 1), while the optimal pH ranges for bacterial and fungal communities are 4–7 and 5–9, respectively [74], thus soil bacteria were more susceptible to pH variations compared to fungi across different wetlands. Soil pH is known to significantly impact membrane-bound proton pumps and the stability of proteins [75], thereby exerting a direct physiological constraint on specific bacteria (Chloroflexi (*SBR1031*, *KD4-96*), Firmicutes (*Bacillus*, *Aliifodinibius*, *Brevibacillus*)), the distribution patterns of these specific bacterial phyla and genus across pH gradient were the same as their pH responses were observed in previous studies [19,76].

In addition, salinity was also an indicator of influence on bacterial community structure (Figure 4A, Table S4), previous studies have consistently supported this conclusion [57,77,78]. Increasing salt concentrations in the environment elevate the osmolarity outside the microbial cell and limit microbial activity [79]. Moreover, the augmentation of salt ions in soil may result in detrimental impacts on microorganisms, as demonstrated by Macêdo [80]. Furthermore, distinct bacterial communities have displayed varying degrees of tolerance towards specific ionic toxicity [78]. In this study, we found that Acidobacteria (*Subgroup_6*, *Subgroup_17*) were significantly negatively correlated with salinity (Figure 5A, Tables S6 and S7), which were adaptable in the plots of *Phragmites australis* community with lower salinity, which was consistent with the results of a previous study [2].

### 4.3. Soil Nutrients and Salinity Determine Fungal Diversity Across Different Wetlands

Soil fungi, notably saprotrophic fungi and mycorrhizal fungi are essential components in the ecosystem, acting as decomposers and forming symbiotic relationships with plants, contributing significantly to the nutrient cycling processes within soils [81,82]. In contrast to bacteria, the diversity and community structures of soil fungi were primarily affected by soil nutrient levels (e.g., TP, AP, and TN) and soil salinity (conductivity) in this study. Our results showed that *Spartina alterniflora* communities closest to the ocean had the highest index of fungal diversity (Figure 2A, Table S1). Soil fungal richness and diversity responded positively with AP and TP, respectively (Table 3). Similar observations were reported by Wang et al. [83], which found that *Spartina alterniflora* invasion significantly increased the amounts of soil AP, and this may be related to the well-developed root tissue of *Spartina alterniflora* and its exudates. Phosphorus is a necessary nutrient element for fungal growth and promotes the synthesis of fungal DNA, RNA, functional enzymes, and cell walls [84]. Previous research has indicated that the predominant soil fungal phyla are notably impacted by fluctuations in TP content [85]. Adequate soil phosphorus levels can enhance the abundance and activity of specific soil fungal taxa.

Ascomycota was the dominant fungal phylum in our study. This aligns with previous studies indicating that the most prevalent fungi in wetland ecosystems are Ascomycota and Basidiomycota [86]. Saprophytic fungi belong to the Ascomycota phylum and play a crucial role in decomposing lignin and keratin in the soil, facilitating soil nutrient transformation and cycling, and enhancing soil quality [87]. Furthermore, TN was found to be the predominant indicator of the variation in fungal community structure across different wetlands in this study (Figure 4B, Table S5). In their role as decomposers of organic matter and symbionts of plants, soil fungi play a crucial part in the nitrogen cycling process within the soil [88]. Previous studies demonstrated that soil fungal communities are involved in the regulation of the soil N cycle, on account of their ability to adapt to a wide variety of microsites, and the secretion of exoenzymes that depolymerize N-containing compounds [89]. Our results showed that Chytridiomycota and Mortierellomycota were positively correlated with TN (Figure 5B, Table S6). The nitophilic nature of fungal species may potentially support their growth, and N may improve the growth and competitive ability of fungal taxa [90]. In addition, we found that Basidiomycota (*Cystofilobasidium* and *Vishniacozyma*) and Glomeromycota were negatively correlated with TN (Figure 5B, Table S6), this was consistent with previous research by Yu et al. [91], which revealed that there is a reduction in the prevalence of Basidiomycota (including various ectomycorrhizal fungi) and Glomeromycota (arbuscular mycorrhizal fungi) following the addition of nitrogen. High N availability leads to a decrease in plants depending on mycorrhizal fungi for nitrogen acquisition, consequently leading to a reduction in the allocation of photosynthetic carbon from the aboveground plant parts to the belowground parts. The depletion of this vital energy source has the potential to impact the symbiotic relationship between plants and mycorrhizal fungi, potentially resulting in a reduction in mycorrhizal taxa [92]. This means that N content may act as an environmental filter to select specific fungal groups across wetlands [93].

Furthermore, soil salinity was another important factor that affected the structure of fungal communities (Figure 4B, Table S5). This is in agreement with other studies [94,95], where the salinity changed the fungal community structure because of the difference in their tolerance to salinity. There was a significant correlation between Ascomycota (*Hortaea*, *Wallrothiella*) and salinity content in our study (Figure 5B, Tables S6 and S7), because Ascomycetes had a wide range of soil adaptation and were suitable for survival under saline conditions, and was consistent with previous research [77,96]. Basidiomycota, Chytridiomycota, and Mortierellomycota have a significant negative correlation with salinity (Figure 5B, Table S6). Salinity may increase the osmolarity outside the fungal cell in high-salt soils, limiting fungal activity [79]. In addition, Krishnamoorthy et al. [97] found that high salinity levels could inhibit the hyphal growth of some mycorrhizal fungi, this may demonstrate the negative influence of salinity on soil fungi.

No significant correlations were detected between the relative abundance of Glomeromycota (arbuscular mycorrhizal fungi, AMF) and soil pH/soil phosphorus content. This finding contrasts with previous studies that suggested the negative impact of pH on arbuscular mycorrhizal colonization rates [98,99]. While other studies have not identified this correlation, they have indicated that soil pH is one of the primary factors influencing AMF community composition [100,101,102]. This may be because different AMF species respond differently to soil pH [103], which in turn affects the overall performance of AMF to soil pH. Meanwhile, soil phosphorus has historically been the focus of AM function and derived plant benefit, due to its scarcity and limited mobility in many ecosystems [104,105], but our findings indicate that soil phosphorus did not significantly affect the relative abundance of AMF. This may be attributed to the relatively high availability of soil phosphorus present in the studied wetlands [91], which potentially led to a diminished reliance of plants on mycorrhizal symbiotic relationships [105].

## 5. Conclusions

This study investigated the diversity patterns and drivers of soil bacterial and fungal diversities across different wetlands in the largest muddy coastal wetland in China. The results suggested that soil fungal diversity exhibited a significant decrease in correlation to the proximity to the coastline, no significant differences were observed in bacterial diversity across different wetlands. It was also evident that soil bacterial and fungal community compositions were both distinctly separated among the different wetlands. Soil nutrients (phosphorus and nitrogen) and salinity were predominant in predicting the variations in fungal diversity and community structure across different wetlands. Soil organic carbon, pH, and salinity were the driving factors of bacterial community structure across different wetlands. This study illuminated the imperative need to explore the different responses of soil microbial diversities to the changing environments, which has important implications for maintaining the ecological balance and functional stability of wetland ecosystems.

## Figures and Tables

**Table 1:** Soil properties across different wetlands in the Tiaozini coastal wetland. SA1, plots of *Spartina alterniflora*; SG2, plots of *Suaeda glauca*; SG3, additional plots of *Suaeda glauca*; PA4, plots of *Phragmites australis*. Soil pH (pH); moisture content (MC); available phosphorus (AP); total phosphorus (TP); ammonia (NH_4_^+^); nitrate (NO_3_^−^); soil organic carbon (SOC); total carbon (TC); total nitrogen (TN). Values indicate means ± SE (*n* = 4). Different letters indicate significant differences between different plots (Tukey’s HSD, *p* < 0.05).

Variables	SA1	SG2	SG3	PA4
Conductivity (mS/cm)	4.64 ± 0.21 c	7.21 ± 0.31 a	6.02 ± 0.08 b	3.03 ± 0.12 d
pH	8.45 ± 0.03 b	7.90 ± 0.02 d	8.69 ± 0.01 a	8.08 ± 0.03 c
MC (%)	71.48 ± 0.42 a	30.97 ± 0.46 c	22.25 ± 0.20 d	37.67 ± 0.20 b
AP (mg/kg)	17.53 ± 0.29 b	15.93 ± 0.05 c	18.99 ± 0.17 a	15.70 ± 0.06 c
TP (mg/kg)	111.07 ± 0.51 a	109.36 ± 0.75 a	102.20 ± 0.79 b	103.45 ± 0.71 b
NH_4_^+^ (mg/kg)	1.77 ± 0.07 a	0.67 ± 0.06 d	1.06 ± 0.05 c	1.30 ± 0.03 b
NO_3_^−^ (mg/kg)	1.21 ± 0.07 a	0.89 ± 0.03 c	1.04 ± 0.04 b	0.74 ± 0.03 d
SOC (g/kg)	12.71 ± 0.28 a	1.58 ± 0.34 d	2.84 ± 0.15 c	3.47 ± 0.25 b
TC (g/kg)	24.83 ± 0.69 a	10.80 ± 0.20 d	12.70 ± 0.26 c	15.45 ± 0.18 b
TN (g/kg)	2.93 ± 0.08 a	2.08 ± 0.05 b	1.87 ± 0.10 b	2.10 ± 0.04 b

**Table 2:** Pearson correlation between microbial alpha-diversity indices and soil properties. Alpha-diversity indices were based on ASVs richness (Observed_species index), diversity (Shannon index). Bold *p*-values indicate statistical significance (*p* < 0.05).

Variables	Bacteria		Fungi	
	Observed_Species	Shannon	Observed_Species	Shannon
Conductivity	−0.191	−0.104	0.197	**0.584**
pH	0.016	−0.05	0.259	0.066
MC	**0.586**	0.372	0.186	0.167
AP	−0.162	−0.107	**0.384**	0.482
TP	0.461	0.495	0.303	**0.635**
NH_4_^+^	0.375	0.107	0.194	−0.032
NO_3_^−^	0.065	−0.064	0.101	0.508
TC	**0.507**	0.123	0.320	0.085
TN	**0.525**	0.287	0.339	0.297
SOC	0.491	0.016	0.271	0.005

**Table 3:** Best AIC-selected linear regression model explaining the microbial alpha-diversity across different wetlands in the Tiaozini coastal wetland. Bold *p*-values indicate statistical significance (*p* < 0.05); RI, relative importance.

	Type	Predictor Variables	Slope (SE)	t-Value	*p*	RI
Bacteria	Observed_species	(intercept)	−5.733 × 10^−17^ (0.219)	0.000	1.000	
		MC	0.881 (0.384)	3.331	**0.037**	
	Shannon	(intercept)	−0.000 (0.222)	−0.002	0.999	
		TP	0.495 (0.241)	2.051	0.061	
Fungi	Observed_species	(intercept)	−2.897 (0.282)	−1.000	1.000	
		AP	0.262 (0.162)	4.246	**0.027**	
	Shannon	(intercept)	−0.000 (0.129)	−0.003	0.998	
		TP	0.526 (0.148)	3.559	**0.005**	0.336
		conductivity	0.575 (0.206)	2.748	**0.019**	0.269

## Data Availability

The datasets analysed during the current study are available from the corresponding author on reasonable request.

## Supplementary Materials

The following supporting information can be downloaded at: https://www.mdpi.com/article/10.3390/jof10110770/s1, Table S1: Mean values of bacterial and fungal alpha diversity indices, ASVs and Nonsingleton across different wetlands in the Tiaozini coastal wetland. Alpha-diversity indices were based on ASVs richness and diversity. SA1, *Spartina alterniflora*; SG2, *Suaeda glauca*; SG3, additional plots of *Suaeda glauca*; PA4, *Phragmites australis*. Values indicate means ± SE (n = 4). Different letters indicate significant differences between different plots (Tukey’s HSD, P < 0.05); Table S2: The relative abundance of the main bacterial and fungal phyla in the Tiaozini coastal wetland. Values indicate means ± SE (n = 4). Bold *p*-values indicate significant differences between different plots (Kruskal-Wallis test, *p* < 0.05). Letter a indicates statistical difference compared with SA1, letter b indicates statistical difference compared with SG2, letter c indicates statistical difference compared with SG3; Table S3: Relative abundance of the main bacterial and fungal genera in the Tiaozini coastal wetland. Values indicate means ± SE (n = 4). Bold *p* -values indicate significant differences between different plots (Kruskal-Wallis test, *p* < 0.05). Letter a indicates statistical difference compared with SA1, letter b indicates statistical difference compared with SG2, letter c indicates statistical difference compared with SG3; Table S4: CCA analysis to determine the influence of soil properties on soil bacterial community structure across different wetlands in the Tiaozini coastal wetland (n = 4); Table S5: CCA analysis to determine the influence of soil properties on soil fungal community structure across different wetlands in the Tiaozini coastal wetland (n = 4); Table S6: Spearman correlations between the relative abundance of main bacterial and fungal phyla and environmental factors across different wetlands in the Tiaozini coastal wetland. Soil pH (pH); moisture content (MC); available phosphorus (AP); total phosphorus (TP); ammonia (NH_4_^+^); nitrate (NO_3_^−^); soil organic carbon (SOC); total carbon (TC); total nitrogen (TN). Bold *p* -values indicate statistical significance (*p* < 0.05); Table S7: Spearman correlations between the relative abundance of main bacterial and fungal genus and environmental factors across different wetlands in the Tiaozini coastal wetland. Soil pH (pH); moisture content (MC); available phosphorus (AP); total phosphorus (TP); ammonia (NH_4_^+^); nitrate (NO_3_^−^); soil organic carbon (SOC); total carbon (TC); total nitrogen (TN). Bold *p*-values indicate statistical significance (*p* < 0.05).
